# *Citrus sinensis* and *Vitis vinifera* Protect Cardiomyocytes from Doxorubicin-Induced Oxidative Stress: Evaluation of Onconutraceutical Potential of Vegetable Smoothies

**DOI:** 10.3390/antiox9050378

**Published:** 2020-05-02

**Authors:** Giacomo Pepe, Emanuela Salviati, Shara Francesca Rapa, Carmine Ostacolo, Stella Cascioferro, Michele Manfra, Giuseppina Autore, Stefania Marzocco, Pietro Campiglia

**Affiliations:** 1 Department of Pharmacy, University of Salerno, 84084 Fisciano, Italy; gipepe@unisa.it (G.P.); esalviati@unisa.it (E.S.); srapa@unisa.it (S.F.R.); autore@unisa.it (G.A.); 2 PhD Program in Drug Discovery and Development, University of Salerno, 84084 Fisciano, Italy; 3 Department of Pharmacy, University of Naples Federico II, 80131 Naples, Italy; ostacolo@unina.it; 4 Dipartimento di Scienze e Tecnologie Biologiche, Chimiche e Farmaceutiche (STEBICEF), University of Palermo, 90123 Palermo, Italy; stellamaria.cascioferro@unipa.it; 5 Department of Science, University of Basilicata, Viale dell’Ateneo Lucano 10, 85100 Potenza, Italy; michele.manfra@unibas.it; 6 European Biomedical Research Institute of Salerno, 84125 Salerno, Italy

**Keywords:** adjuvant therapy, anthracyclines, antioxidants, apoptosis, cardiotoxicity, functional foods, onconutraceutical, oxidative stress, polyphenols

## Abstract

The interest towards nutraceuticals able to counteract drug side effects is continuously growing in current chemotherapeutic protocols. In the present study, we demonstrated that smoothies containing mixtures of *Citrus sinensis* and *Vitis vinifera* L. cv. Aglianico N, two typical fruits of the Mediterranean diet, possess bioactive polyphenols that protect cardiomyocytes against doxorubicin-induced oxidative stress. The polyphenolic extracts isolated from *Citrus sinensis-* and *Vitis vinifera*-based functional smoothies were deeply characterized by Liquid Chromatography-Mass Spectrometry methods. Subsequently, the functional smoothies and relative mixtures were tested to verify their ability to affect cellular viability and oxidative stress parameters in embryonic cardiomyocyte cells (H9c2), and human breast adenocarcinoma cell line (MCF-7) exposed to doxorubicin. Interestingly, we found that the mix resulting from *Citrus sinensis* and *Vitis vinifera* association in ratio 1:1 was able to reduce cardiomyocytes damage induced by anthracyclines, without significantly interfering with the pro-apoptotic activity of the drug on breast cancer cells. These results point out the potential use of vegetable smoothies as adjuvants functional foods for chemotherapeutic anticancer protocols.

## 1. Introduction

Anthracyclines are chemotherapeutic agents employed in the treatment of a wide range of cancer such as breast cancer, leukemia, lymphoma, lung cancer, multiple myeloma, and sarcoma. The clinical use of anthracyclines, in particular doxorubicin, is well-known to be limited by the cumulative dose-related toxicity, the reduction in left ventricular ejection fraction (LVEF), and the irreversible cardiotoxicity [1]. In particular, cardiotoxicity usually occurs one year after the completion of chemotherapy. Moreover, a rare form of acute cardiotoxicity, characterized by pericarditis and arrhythmias, has been described [2]. The heart is a target organ of oxidative stress-related injuries because of its very high energetic metabolic demand and lower levels of antioxidant agents and enzymes [3]. The chronic cardiotoxicity of doxorubicin induces cardiomyocyte death through free radical generation caused by mitochondrial Nicotinamide adenine dinucleotide (NADH) dehydrogenase-catalyzed reduction of anthracycline and by interference with iron metabolism catalyzing the Fenton reaction [4].

Many studies have demonstrated that healthy lifestyle and diet can help to prevent the insurgence of chronic pathologies such as cardiovascular and neurodegenerative diseases, diabetes, and cancer [5]. Nowadays, high attention has been addressed to natural components in fruits and vegetables with potential antioxidant, anti-inflammatory, anticarcinogenic and antimutagenic properties [6,7,8,9]. In particular, some scientific evidence described the potential health effects that dietary polyphenols are able to exercise on biological systems through antioxidant, cardioprotective, anti-inflammatory and anti-proliferative activities [10]. The consumption of dietary supplements and nutraceuticals in cancer therapy is mainly aimed at chemoprevention to reduce the drug resistance, to the identification of synergistic effects with anticancer drugs in order to decrease drug concentrations and, consequently, the side effects associated with current cancer treatments. In fact, chemotherapy protocols including surgery, pharmacotherapy, radiotherapy, and biologically based therapies, have unintended side effects, which compromise both health and well-being of patients [11]. In this regard, dietary polyphenols have been widely demonstrated to be able to not only reduce oxidative and inflammatory stress typical of the onset and development of cancer, but also to counteract the side effects associated with drug therapy [12,13].

Different species of plants are able to protect against cancer including *Vitis vinifera* and *Citrus sinensis* [14]. Red grape juice (*Vitis vinifera* L. cv. Aglianico N) demonstrated an appreciable direct radical-scavenging activity, able to contrast the doxorubicin-induced oxidative stress in cardiac-derived myocytes, decreasing ROS levels and depressing caspase-3 activity [15]. It has been also reported the antioxidant and anti-inflammatory potential of *Citrus sinensis*. The methanolic extract was able to reduce macrophages pro-inflammatory mediators and different cytokines. Furthermore, *Citrus sinensis* polyphenols have been shown to have antioxidant effect by reducing reactive oxygen species (ROS) and increasing cytoprotective enzymes expression [16]. In this regard, in the present study, we evaluated the ability of *Vitis vinifera* and *Citrus sinensis* smoothies, and relative mixes, to inhibit and counteract the doxorubicin-induced oxidative stress in embryonic rat heart-derived cells (H9c2). Moreover, the antiproliferative activity of the smoothies in human breast adenocarcinoma cell line (MCF-7) exposed to the anthracycline was also evaluated. 

## 2. Materials and Methods 

### 2.1. Reagents and Standards

Ultra pure water (H_2_O) was obtained by a Milli-Q Direct 8 system (Millipore, Milan, Italy), acetonitrile (ACN), formic acid (HCOOH) and acetic acid (CH_3_COOH) LC-MS grade were purchased by Sigma-Aldrich (Milan, Italy). Polyphenol standards (apigenin 6,8-C-β-d-glucopyranoside, naringenin, narirutin, hesperidin, didymin, and malvidin 3-*O*-glucoside chloride) were purchased by Extrasynthese (Lyon, France). Unless otherwise specified, all reagents and compounds were purchased from Sigma Chemicals Company (Sigma, Milan, Italy).

### 2.2. Sample Preparation

Grape (*Vitis vinifera* L. cv. Aglianico N) and orange (*Citrus sinensis*) smoothies were kindly donated by the company DO.DA.CO. Srl, (Scafati, Salerno, Italy). The smoothies were lyophilized for 72 h, at −52 °C, setting the vacuum to 0.100 mBar (LyoQuest −55, Telstar Technologies, Terrassa, Spain) obtaining from 100 g of orange and grape smoothie, respectively, 10.22 ± 0.05 g and 27.47 ± 0.17 g of dried sample. In order to extract polyphenolic compounds, 5 g of lyophilized orange smoothie was treated with 30 mL of methanol/water (80:20 v/v) and kept under stirring for 1 h at room temperature while to extract anthocyanins, the lyophilized grape smoothie was treated with 40 mL of methanol plus 0.1% HCl (v/v) overnight at room temperature [17,18]. Subsequently, the obtained extracts were centrifuged at 6000 rpm at 4 °C, for 10 min (Mikro 220R centrifuge, Hettich, Germany) and then the supernatants were filtered through 0.45 μm nylon membrane filters, analyzed by Reversed-phase-Ultra-high performance liquid chromatography-Tandem mass spectrometry (RP-UHPLC-MS/MS) and then lyophilized. For the preparation of different bioactive mixture, each polyphenolic extract was solubilized in methanol:water (50:50 v/v) and mixed in different ratio to obtain 3 different mixture (1:1 w/w; 2:1 w/w Orange:Red grape; 1:2 w/w O:Rg), organic solvent was evaporated under nitrogen flow and finally the solution was lyophilized.

### 2.3. Qualitative Analysis

#### 2.3.1. Instrumentation

HPLC analyses were performed on a Shimadzu Nexera UHPLC system, consisting of a CBM-20A controller, two LC-30AD dual-plunger parallel-flow pumps, a DGU-20 AR5 degasser, an SPD-M20A photo diode array detector, a CTO-20A column oven, a SIL-30AC autosampler. The instrument was coupled with a hybrid Ion trap-Time of Flight Mass spectrometer (LCMS-IT-TOF, Shimadzu, Kyoto, Japan) equipped with an electrospray source (ESI) operated in negative and positive mode. LC-MS data elaboration was performed by the LCMS solution^®^ software (Version 3.50.346, Shimadzu, Kyoto, Japan).

#### 2.3.2. Chromatographic Conditions: RP-UHPLC-PDA 

For RP-UHPLC analyses a Kinetex^®^ EVO C18 150 mm × 2.1 mm, 2.6 µm (100 Å) (L × I.D, particle size, Phenomenex^®^, Bologna, Italy) column was employed at a flow rate of 0.5 mL/min. The separation of polyphenols isolated from both smoothies was carried out with the following parameters: mobile phase was: A) 0.1% CH_3_COOH in H_2_O v/v, B) ACN plus 0.1% CH_3_COOH, gradient: 0–2.0 min, 0–10% B; 2–20.0 min, 10–20% B; 20–21.0 min, 20–70% B; 21–21.50 min, 70–95% B. For the analysis of the anthocyanins extracted from grape smoothies, the mobile phases consisted of A) 0.1% TFA in H_2_O and B) ACN plus 0.1% TFA. Analysis was performed in gradient as follows: 0–10.0 min, 5–30% B; 10–13.0 min, 30–70% B; 13–14.0 min, 60–98% B. Column oven was set to 45 °C, 2 µL of each extract were injected. PDA detection parameters were sampling rate 12 Hz, time constant 0.160 s, and chromatograms were extracted at 280, 330 and 520 nm.

#### 2.3.3. LCMS-IT-TOF Parameters

Ultra-high performance liquid chromatography (UHPLC) system was coupled online to IT-TOF instrument, in RP-UHPLC flow rate from liquid chromatography (LC) was split by a Tee union prior the Electrospray ionization (ESI) source. Mass spectrometry (MS) ionization was operated in negative mode for the polyphenols analysis, while in positive mode for the anthocyanins analysis isolated from grape smoothies. Detection was performed as follows: curve desolvation line (CDL), 250 °C; Block Heater, 250 °C; Nebulizing and Drying gas, 1.5 and 10 L/min; ESI-Capillary Voltage, −3.5 kV; ESI + Capillary Voltage, +4.5 kV. MS range, *m/z* 150–1500; ion accumulation time, 25 ms; ion trap repeat, 3. MS/MS was performed in data-dependent acquisition (DDA), precursor ions selection was based on the base peak chromatogram (BPC) intensity of 150.000. Collision induced dissociation (CID), 50%; ion trap repeat, 1. TOF accuracy and resolution were adjusted injecting a Sodium trifluoroacetate (NaTFA) solution prior the analysis.

The identification of bioactive compound was based on accurate MS and MS/MS spectra, UV absorbance, comparison of available standards and MS database searching. “Formula Predictor” software (Shimadzu, Kyoto, Japan) was used for the prediction of the molecular formula, using the following settings: maximum deviation from mass accuracy: 5 ppm, fragment ion information, and nitrogen rule.

### 2.4. Quantitative Analysis

Apigenin 6,8-C-β-d-glucopyranoside, naringenin, narirutin, hesperidin, and didymin were selected as external standards for the quantification of the polyphenols isolated from the orange smoothie. The quantitative analysis of the anthocyanins extracted from grape smoothie was performed using malvidin 3-*O*-glucoside as external standard. Stock solutions (1 mg/mL) were prepared in methanol and the calibration curves were obtained in a concentration range of 1–200 μg/mL with five concentration levels and performing triplicate analysis for each level. The linear regression was used to generate the calibration curve that correlates peak area versus analyte concentrations with values of correlation coefficient *R*^2^ ≥ 0.999. The peak areas were converted to the corresponding concentrations (µg/mL) and the amount of the compounds in the sample was expressed as milligrams per 100 g of extract.

### 2.5. Biological Evaluation

#### 2.5.1. Cell Culture

Embryonic rat cardiomyocytes (H9c2) and human breast cancer cell line (MCF-7) were purchased from American Type Culture Collection (Rockville, MD, USA). Cells were grown adjacent to Petri dishes with Dulbecco’s modified Eagle’s medium (DMEM), 10% (v/v) fetal bovine serum (FBS), 2 mM L-glutamine and 100 U/mL penicillin and 100 mg/mL streptomycin at 37 °C in a 5% CO_2_ atmosphere. 

#### 2.5.2. Cell Treatment

H9c2 and MCF-7 cells were plated in 96 or 24 well plates and allowed to adhere for 24 h. Thereafter, the medium was replaced with pure fresh medium or containing serial dilutions of tested compounds (1–25 μg/mL) for 1 h, and then co-exposed to a final concentration of Doxorubicin (DOXO; 5 μM) for further 24 h, in order to evaluate, respectively, the cardioprotective effect of orange and red grape smoothies and their respective mixtures, in ratio 1:1, 1:2 and 2:1 [O:RG w/w], on cardiomyocytes and the cellular viability in human breast adenocarcinoma cell line.

#### 2.5.3. Cell Viability

H9c2 (5 × 10^3^/well) and MCF-7 cells (2.0 × 10^4^/well) were plated on 96-well plates and allowed to adhere for 24 h. Thereafter, the medium was replaced with fresh medium alone or containing serial dilutions of orange and red grape smoothies and their respective mixtures in ratio 1:1, 1:2 and 2:1 [O:RG w/w] (1–25 μg/mL), and incubation was performed for 24 h. Cell viability was assessed through 3-(4,5-dimethyltiazol-2yl)-2,5-phenyl-2H-tetrazolium bromide (MTT) assay as previously reported [19]. Briefly, 25 μL of MTT (5 mg/mL) was added and the cells were incubated for an additional 3 h. Thereafter, cells were lysed and the dark blue crystals solubilized with 100 μL of a solution containing 50% (mL L^−1^) N,N-dimethylformamide, 20% (mL L^−1^) sodium dodecyl sulfate (SDS) with an adjusted pH of 4.5. The optical density (OD) of each well was measured with a microplate spectrophotometer Titertek (Dasit, Cornaredo, Milan, Italy), equipped with a 620 nm filter. Cell viability in response to treatment was calculated as:

% cell viability = (OD treated/OD control) × 100(1)

#### 2.5.4. Measurement of Intracellular ROS Release

ROS formation was evaluated by means of the probe 2′,7′-dichlorofluorescin-diacetate (H_2_DCF-DA). H_2_DCF-DA is a non-fluorescent permeant molecule that passively diffuses into cells, where the acetates are cleaved by intracellular esterases to form H_2_DCF, and thereby traps it within the cell. In the presence of intracellular ROS, H_2_DCF is rapidly oxidized to the highly fluorescent 2′,7′-dichlorofluorescein (DCF). After cell treatment, previously reported, cells were collected, washed twice with phosphate buffer saline (PBS), and then incubated in PBS containing H_2_DCF-DA (10 µM) at 37 °C. After 15 min, fluorescence was evaluated using a fluorescence-activated cell sorting (BD FacsCalibur, Milan, Italy) and elaborated with Cell Quest software, as previously reported [20].

#### 2.5.5. HO-1 and NQO1 Detection by Cytofluorimetry 

H9c2 cardiomyocytes and MCF-7 breast cancer cells were plated into 96-well plates (5 × 10^3^ cells/well and 2.0 × 10^4^ cells/well respectively) and were allowed to grow for 24 h at 37 °C in a 5% CO_2_ atmosphere before experiments. Thereafter, the medium was replaced with fresh medium, and cells were treated with serial dilutions of mix composed by orange and red grape in ratio 1:1 [w/w] (1–25 μg/mL), added 1 h before and simultaneously to DOXO (5 μM), for 24 h. Cells were collected, washed twice with PBS, incubated in Fixing Solution for 20 min at 4 °C, and then incubated in Fix Perm Solution for 30 min at 4 °C. Anti-HO-1 and anti-NQO1 (Santa Cruz Biotechnologies, Dallas, TX, USA) antibodies were added to H9c2 and MCF-7 cells for a further 30 min. The secondary antibody was added in Fix Perm solution and cells were evaluated using a fluorescence-activated cell sorting (FACSscan; Becton Dickinson, Milan, Italy) and elaborated with Cell Quest software, as previously reported [21].

#### 2.5.6. Analysis of Apoptosis

Hypodiploid DNA was analyzed using propidium iodide (PI) staining by flow cytometry [22]. Briefly, H9c2 (1 × 10^3^) and MCF-7 (3.5 × 10^5^) cells were grown in 24-well plates and allowed to adhere. Thereafter cells were synchronised for 24 h in serum-free media before being exposed to mix composed by Orange and Grape in ratio 1:1 [w/w] (1–25 μg/mL) alone and in combination with DOXO (5 μM), for further 24 h. Following treatment, culture medium was removed, cells washed once with PBS, and then resuspended in 500 µL of a solution containing 0.1% (w/v) sodium citrate and 50 μg/mL PI. Culture medium and PBS were centrifuged and cell pellets were pooled with cell suspension to retain both dead and living cells for analysis. After incubation at 4 °C for 30 min in the dark, cell nuclei were analyzed with a Becton Dickinson FACScan flow cytometer using the CellQuest program.

### 2.6. Data Analysis 

Data are reported as mean ± standard error of the mean (s.e.m.) values of at least three independent experiments each in triplicate. Statistical analysis was performed by analysis of variance test, and multiple comparisons were made by Bonferroni’s test by using Prism 5 (GraphPad Software, San Diego, CA, USA). p-values smaller than 0.05 were considered significant.

## 3. Results

### 3.1. Qualitative and Quantitative Profiles of Smoothie Extracts

**Figure 1:**
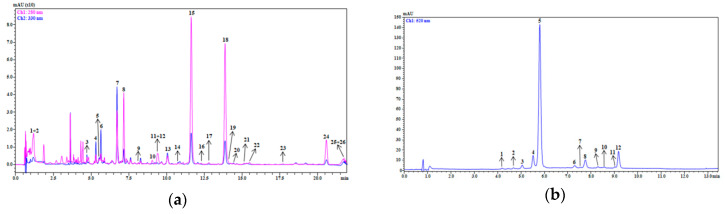
Chromatogram profiles (λ: 280 nm and λ: 330 nm; pink and blue lines, respectively) of polyphenols isolated from *Citrus sinensis* smoothie (**a**) and anthocyanins (λ: 520 nm) extracted from *Vitis vinifera* L. cv. Aglianico N smoothie (**b**). (**a**): #1: quinic acid; #2: caffeoylquinic acid; #3: 5-*p*-coumaroyl-hexoside acid; #4: ferulic acid 4-*O*-glucoside; #5: ferulic acid hexoside; #6: quercetin 3-*O*-glucosyl-rhamnosyl-hexoside; #7: vicenin II (apigenin 6,8-C-β-d-glucopyranoside); #8: naringenin hexoside; #9: lucenin 2-4′-methyl-ether; #10: eriocitrin; #11: quercetin 3-*O*-glucosyl-rhamnosyl-hexoside; #12: quercetin-3-*O*-hexoside; #13: apigenin 7-*O*-apiosyl-glucoside; #14: rutin; #15: narirutin; #16: naringenin 3-*O*-glucosyl-rhamnosyl-hexoside; #17: naringenin 7-*O*-neohesperidoside; #18: hesperidin; #19: limonin glucoside (limonin 17β-d-glucopyranoside); #20: isorhamnetin 3-*O*-rutinoside; #21: hesperidin isomer; #22: hesperidin isomer; #23: deacetyl-nomilinic acid glycoside; # 24: didymin; #25: nomilin glucoside; #26: nomilinic acid-glycoside. (**b**): #1: delphinidin 3 *O*-glucoside; #2: cyanidin 3 *O*-glucoside; #3: petunidin 3 *O*-glucoside; #4: peonidin 3 *O*-glucoside; #5: malvidin 3 *O*-glucoside; #6: petunidin 3 *O*-(6”acetyl) glucoside; #7: peonidin 3 *O*-(6”acetyl) glucoside; #8: malvidin 3 *O*-(6”acetyl) glucoside; #9: malvidin 3 *O*-(6”-*p*-caffeoyl) glucoside; #10: petunidin 3-(6-coumaroyl) *O*-glucoside; #11: peonidin 3-(6-coumaroyl) *O*-glucoside; #12: malvidin 3 *O*-(6”-*p*-coumaroil) glucoside.

Qualitative and quantitative analysis of bioactive compounds were carried out by UHPLC-PDA-MS/MS analysis. Figure 1 shows the chromatographic profiles of polyphenols and anthocyanins isolated from orange (1a) and red grape (1b) smoothies, respectively. The complete lists of polyphenolic compounds tentatively identified are reported in Tables S1 and S2.

Among the identified compounds in the orange smoothie, different classes were present, in particular: hydroxycinnamic acids (5), flavonols (5), flavones (4), and flavanones (8). Hydroxycinnamic acids eluted in first part of the chromatogram followed by flavonoids. In detail, the compound 7 showed MS/MS fragment ions at *m*/*z* 473 [M−H−120]^−^ and 503 [M−H−90]^−^, probably derived from the sequential loss of two hexose moieties specific of C-glycoside, so it was tentatively identified as apigenin 6,8-C-β-d-glucopyranoside (Figure S1a). Peaks 15 and 18 were the most intense in the chromatographic profile. The peak 15 showed a precursor ion at *m*/*z* 579 and provided the fragment ion at *m*/*z* 271, deriving from the loss of the di-hexoside moiety and from the rearrangement of the aglycone naringenin, so it was proposed as narirutin (Figure S1b). The peak 18 at *m*/*z* 609, instead, showed a fragment ion at *m*/*z* 301, which assumes the loss of a hexose glycoside moiety and the rearrangement of the deprotonated aglycone hesperitin, leading to its tentative identification as hesperidin (Figure S1c). Among limonoid glycosides, compounds 23 and 26 had [M−H]^−^ 669 *m*/*z* and 711 *m*/*z*, respectively, and they were characterized by the same fragment patterns with ions at *m*/*z* 609 and *m*/*z* 607, probably derived from the loss of an acetyl moiety. They were tentatively identified as deacetyl-nomilinic acid glycoside and nomilinic acid glycoside (Figure S1d,e).

In the grape smoothie extract, the peak having [M−H]^−^ 493 *m*/*z* was the most intense. Its fragmentation pattern showed a fragment ion at *m*/*z* 331, deriving from the loss of hexose and resulting in the deprotonated aglycone malvidin. Therefore, it was proposed as malvidin 3-*O*-glucoside and its identity was further confirmed by the comparison with the available standard. The quantitative analysis of grape smoothie extract showed that the most abundant compound was malvidin 3-*O*-glucoside (#5b, 552.25 ± 1.70 mg 100 g^−1^), while the orange smoothie extract was rich in narirutin (#15a, 213.14 ± 3.32 mg 100 g^−1^), hesperidin (#18a, 188.02 ± 3.25 mg 100 g^−1^), apigenin 6,8-C-β-d-glucopyranoside (#7a, 62.04 ± 1.01 mg 100 g^−1^), naringenin hexoside (#8a, 38.05 ± 0.59 mg 100 g^−1^) and didymin (#24a, 57.92 ± 1.03 mg 100 g^−1^).

### 3.2. Effect of Orange and Grape Smoothies and Their Respective Mixtures, on H9c2 and MCF-7 Cells Viability

In order to evaluate the viability of cells trated with orange, red grape smoothies, and relative mixtures (1–25 μg/mL) for 24 h, MTT assay was performed. The results indicated that the tested extracts, as well as the relative mixtures, alone do not significantly affect the vitality of H9c2 cells (Figure S2a,b). Also MCF-7 cells viability was not significantly affected by tested extracts and relative mixtures (Figure S2c,d).

**Figure 2:**
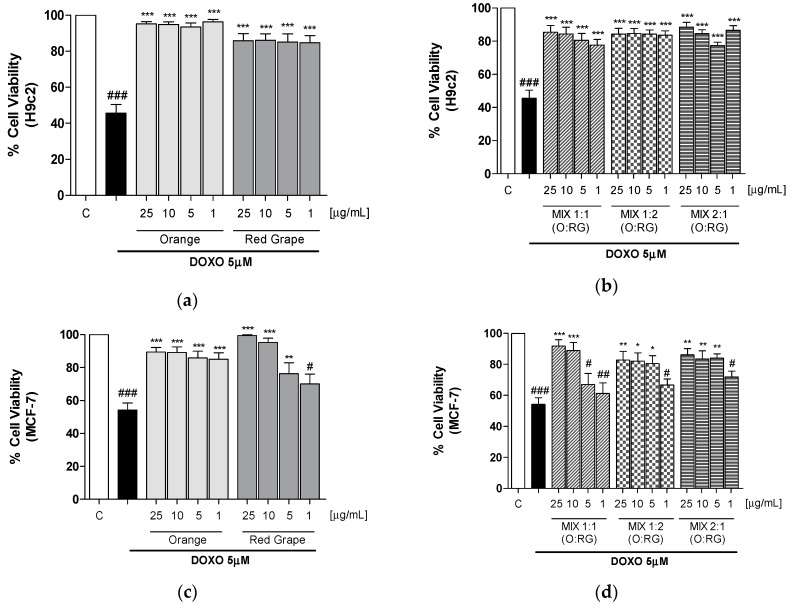
Effect of orange and red grapes extracts, and the 3 relative mixtures (1–25 μg/mL) in combination with DOXO (5 μM) on viability of H9c2 (**a**,**b**) and MCF-7 (**c**,**d**) cells, evaluated by MTT assay. Data are expressed as mean ± S.E.M. of % of cell viability. ***, ** and * denote respectively *p* < 0.001, *p* < 0.01 and *p* < 0.05 vs DOXO alone. ###, ## and # denote respectively *p* < 0.001, *p* < 0.01, and *p* < 0.05 vs C.

In another set of experiments, cells were treated with orange and red grape extracts, and the 3 relative mixtures, in combination with doxorubicin (DOXO; 5 μM). The results indicated that tested extracts reduced the antiproliferative activity induced by doxorubicin in H9c2 at all tested concentrations (*p* < 0.001 vs DOXO; Figure 2a,b). Interestingly, the doxorubicin antiproliferative activity on MCF-7 cells was affected at all the concentrations tested from orange and at the three higher concentrations for red grape (*p* < 0.05 vs DOXO; Figure 2c,d). The reduction in doxorubicin-induced antiproliferative activity by the extracts was more pronounced in H9c2 than in MCF-7 cells. Similarly, the mixtures evaluation indicated that while a significant inhibition of the antiproliferative activity is induced in H9c2 at all the tested concentrations (*p* < 0.001 vs DOXO Figure 2b), on MCF-7 a significant inhibition was observed at the two higher concentrations for MIX 1:1 and at the three higher doses for the other mixtures (*p* < 0.05 vs DOXO Figure 2d).

These results highlighted that, among the tested mixtures, the MIX 1:1 was the most effective in protecting cardiomyocytes from doxorubicin side effects without affecting doxorubicin efficacy on MCF-7 at 5 and 1 μg/mL doses.

### 3.3. Effect of Mix 1:1 Reduced on Doxorubicin-Induced ROS Release in H9c2 and MCF-7 Cells

**Figure 3:**
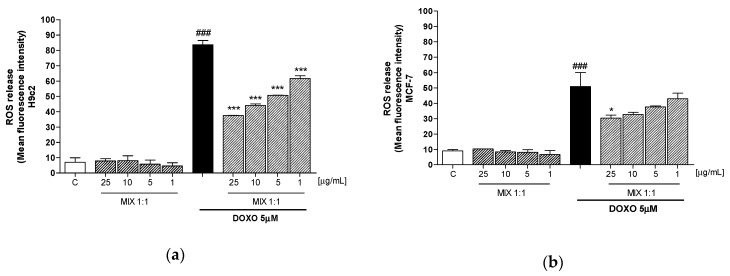
Effect of more effective smoothies’ MIX 1:1 ratio, (1–25 μg/mL), on intracellular ROS release alone and in combination with DOXO (5 μM) in H9c2 (**a**) and MCF-7 (**b**) cells, detected with the probe 2′,7′ dichlorofluorescein-diacetate (H_2_DCF-DA). Data are expressed as mean ± s.e.m. of mean fluorescence intensity. *** and * denote respectively *p* < 0.001 and *p* < 0.05 vs DOXO; ### denotes *p* < 0.001 vs C.

In order to evaluate the mechanism underlying the effects of MIX 1:1 in cardiomyocytes, we analyzed its antioxidant potential, through the evaluation of intracellular ROS release. The Figure 3 shown that smoothies’ MIX 1:1 ratio (1–25 μg/mL), was able to provide concentration-dependant reduction of the doxorubicin-induced ROS release, in H9c2 (*p* < 0.001 vs DOXO; Figure 3a). Contrariwise, in MCF-7 cell line the MIX 1:1 reduced ROS release only at 25 μg/mL (*p* < 0.05 vs DOXO; Figure 3b). These results point out one of the potential mechanism underlying the selective protective efficacy of the MIX 1:1 on cardiomyocytes.

### 3.4. Effect of MIX 1:1 on Doxorubicin-Induced HO-1 and NQO1 Expression

**Figure 4:**
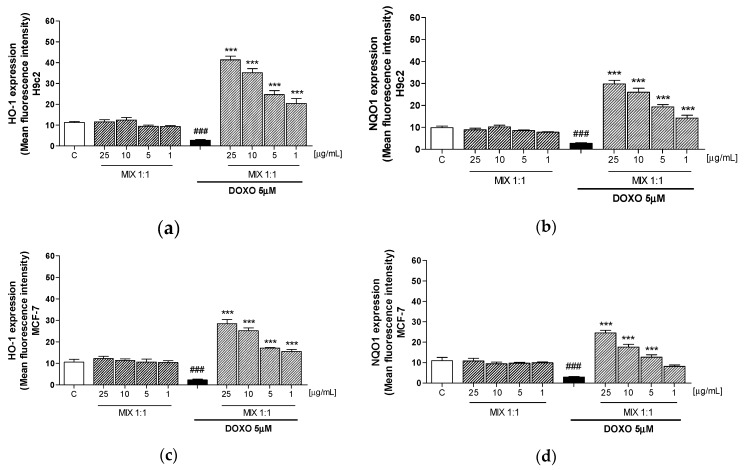
Effect of most effective smoothies’ MIX 1:1 ratio (1–25 μg/mL), on HO-1 and NQO1 expression, alone and in combination with DOXO (5 μM) in H9c2 (**a**,**b**) and MCF-7 (**c**,**d**) cells, evaluated by cytofluorimetric technique. Data are expressed as mean ± s.e.m. of mean fluorescence intensity. *** denotes *p* < 0.001 vs DOXO; ### denotes *p* < 0.001 vs C.

Oxidative stress is due to a disequilibrium between pro-oxidant, such as ROS, and antioxidant factors. In order to evaluate the antioxidant activity of MIX 1:1, we evaluated the expression of cytoprotective factors, such as HO-1 and NQO1, by the cytofluorimetric technique in doxorubicin-treated H9c2 and MCF-7 cells. DOXO decreased the HO-1 and NQO1 expression in H9c2 cells, while producing a significant decreased in MCF-7 cells. Tested mix (25–1 μg/mL) gave a concentration-dependant increase of HO-1 and NQO1 expression in both doxorubicin treated H9c2 (*p* < 0.001 vs DOXO; Figure 4a,b) and MCF-7 cells (*p* < 0.001 vs DOXO; Figure 4c,d). In particular, the increase in HO-1 expression was more pronounced in H9c2 in comparison with MCF-7 cells thus further confirming the major cardioprotective effect of the smoothies’ MIX 1:1.

### 3.5. Effect of MIX 1:1 Reduced Doxorubicin-Induced Apoptosis

**Figure 5:**
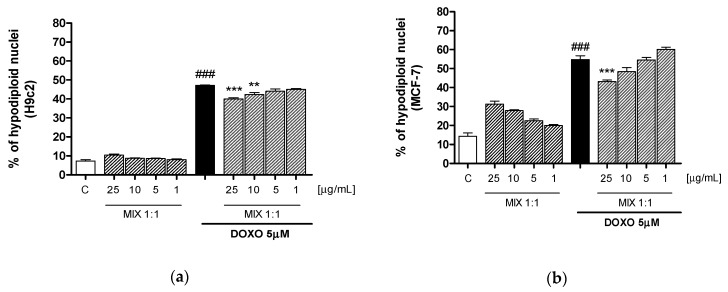
Apoptosis detection by propidium iodide (PI) staining of hypodiploid nuclei after H9c2 (**a**) and MCF-7 (**b**) incubation with MIX 1:1 ratio (1–25 μg/mL), alone and in combination with DOXO (5 μM) for 24 h. Data are expressed as mean ± s.e.m. of % of hypodiploid nuclei. *** and ** denote respectively *p* < 0.001 and *p* < 0.01 vs DOXO; ### denotes *p* < 0.001 vs C.

Apoptosis was evaluated by cytofluorimetric analysis of PI stained hypodiploid nuclei in order to further investigate the biochemical basis for the protective effect of MIX 1:1 on cardiomyocytes and, on the other hand, the retention of the pharmacological effect on MCF-7. Our results indicate that MIX 1:1 (1–25 μg/mL) significantly reduced, at the two highest tested concentrations, doxorubicin-induced apoptosis in H9c2 cells (*p* < 0.01 vs DOXO; Figure 5a) while the same effect was evidenced in MCF-7 breast cancer cells only at 25 μg/mL (*p* < 0.001 vs DOXO; Figure 5b). 

## 4. Discussion

Citrus fruits and red grape juice are considered worldwide as a healthy food due to the high content of bioactive compounds and they are increasingly used in pharmaceutical and nutraceuticals fields for their antioxidant, anti-inflammatory, anti-proliferative and potential anticancer activities [23,24]. In this regard, the use of nutraceutical formulations based on phytochemical extracts both in chemoprevention and as adjuvants in support of pharmacological therapies, is increasingly being proposed [25]. 

Although anthracyclines effectiveness is still a gold standard in the treatment of various cancer, the clinical use of doxorubicin is limited by severe cardiotoxicity [26]. This cytotoxic side effect occurs through multiple mechanisms, which mainly involve doxorubicin accumulation in myocardial mitochondrial membrane and subsequent reactive oxygen species production. In addition, doxorubicin quinone-containing structure is prone to ROS release by redox-cycling reaction that, associated to a reduction in endogenous antioxidants, increase oxidative stress, ultimately leading to myocyte apoptosis [27]. Among several strategies to limit doxorubicin adverse effects, natural polyphenolic compounds are notable as a cardioprotective agent because of the low toxicity and good antioxidant properties through radical-scavenging activity, carbonyl reductase-inhibitory effects, as well as, modulation of endogenous antioxidant enzymes [28,29,30,31]. Apart from their antioxidant effects, also apoptosis modulation is involved in the protective properties of the flavonoids against doxorubicin toxicity [32].

In this work, we evaluated the use of different mixed ratio of commercial nutraceutical smoothies, obtained from *Citrus sinensis* and *Vitis vinifera* fruits, in chemotherapeutics protocols to alleviate anthracyclines adverse effects, but also enhance drugs selectivity, lowering their dosage, without interfere with pharmacological effect on cancer cells. 

*Citrus* flavonoids have been shown to reduce cardiovascular disease prominently due to their antioxidant effects [33]. In this regard, hesperidin, abundant flavanone glycoside present in *citrus* fruits, has been demonstrated to modulate the inflammatory responses and antioxidant status following acute myocardial infarction and could be considered as a promising candidate to alleviate cardiotoxic effect of doxorubicin [34]. In addition, hesperitin, the aglycone of hesperidin, has a cardioprotective effect increasing GHS levels and preventing oxidative DNA damage in rat models [35]. Furthermore, has been reported that Pummelo (*Citrus maxima*) phytocomplex exert protection in cardiomyocytes by reduction of intracellular oxidative stress, maintaining GSH availability, and enhancing GST enzyme activity and expression in rat cardiac cells [36].

Some scientific evidence has attributed to grape proanthocyandins the contribution to iron and calcium levels normalizations and apoptotic signaling pathways regulation in human cardiomyocytes cells subjected to doxorubicin effects [37,38]. However, grape is also a rich source of anthocyanins that possess a good antioxidant activity. Indeed, anthocyanins have gained increasing interest for their protective effect on doxorubicin-induced cardiotoxicity due to the superoxide anion scavenging activity in rat cardiomyocytes [39]. Furthermore, has been recently found that dietary supplementation of bilberry extract protects against doxorubicin-induced cardiotoxicity in rats [40,41]. 

LC-MS analyses were used to assess the stability of the polyphenolic profile in the smoothies. In particular, in the hydro-alcoholic extract of orange smoothie, 22 polyphenols and 4 limonoids glycosides were identified. In accordance with our previous results [8,16], the most abundant compounds were the flavanone narirutin and hesperidin. However, narirutin content was higher in smoothies than fresh juice. The increase may be due to the stage of maturation of the fruits used [42]. Furthermore, the anthocyanin profile of grape remained almost unchanged, and malvidin 3-*O*-glucoside was the main representative anthocyanin. It can be concluded that employment of innovative sterilization and packaging techniques developed and employed by the producer, are able to preserve the organoleptic, nutritional and functional properties of relative fruits, and has no influence on the quantitative and qualitative polyphenolic composition. 

After analytical characterization, the investigated matrices were tested for their ability to reduce the doxorubicin-induced cardiotoxicity in embryonic cardiomyocyte cell lines (H9c2) and for their synergistic role with the anticancer effect of the drug on human breast cancer cell lines (MCF7). 

Initial antiproliferative screening, showed that both the single smoothies and all the possible mix did not influence cellular proliferation, at the concentrations used in this study. The evaluation of antiproliferative activity indicated that both orange and red grape protected cardiomyocytes from doxorubicin, even if this effect was slightly higher for orange. On the other hand, red grape was more active in maintaining the doxorubicin antiproliferative effect on MCF-7 cells but previous experiments indicated also a concentration-related antiproliferative effect of red grape alone on cardiomyocytes (>25 µg/mL; data not shown). In this regard, the employment of mixed phytochemical combination is an attractive option. Thus, we decided to reduce red grape concentration and to add orange in different combinations to identify a mixture able to reduce doxorubicin-induced cardiotoxicity, without altering its therapeutic effect on cancer cells. Moreover, the wide differences in polyphenolic structures among the two matrices could be responsible for different mechanisms of action, integrating the radical scavenging activity with the stimulation of the endogenous antioxidant response [43].

Interestingly, among the tested mixture, the MIX 1:1 was able to protect cardiomyocytes from doxorubicin-induced antiproliferative effect without reducing the effect on MCF-7 cells. Moreover, while red grape alone did not affect doxorubicin activity on MCF-7 cells only at the lower concentration (1 µg/mL), this activity for the MIX 1:1 was observed at the two lowest concentrations (5 and 1 µg/mL) maintaining the same protective effects on cardiomyocytes (Figure 2). This might be due to an optimal balance of antioxidant agent concentration in the MIX 1:1 in comparison with the single smoothies.

To assess the cardioprotective effects of the smoothie mixtures, H9c2 and MCF-7 cells were exposed to different concentrations of orange and red grape mixtures in combination with doxorubicin. Selected concentrations were chosen based on previous investigations, to avoid the pro-oxidant effect of the polyphenols at high doses [18,44]. MIX 1:1 provided an appreciable radical-scavenging activity, significantly reducing ROS release in H9c2 cells at all the tested concentrations, while only at the highest dose in MCF-7 cells. ROS release is one of the main mechanisms triggered by chemotherapy agents to induce cellular death and, on the other hand, able to mediate chemotherapy-associated toxicity in healthy cells/tissue [45]. Therefore, the ability of the MIX 1:1 to differently act on these two cells type points out its potential to reduce the myocardial injury induced by anthracycline, without interfering with its anticancer effect.

Interestingly, we observed that following doxorubicin treatment, H9c2 cells present significantly high ROS levels compared to MCF-7 cells. This may be due to endogenous deficiency in cardiomyocytes of antioxidants and cytoprotective enzymes as HO-1 and NQO1 [3]. These metabolizing enzymes, play a key role both in reducing ROS generation and in the cellular detoxification of highly reactive molecules, generated by the interaction of ROS with biological macromolecules. HO-1 is the primary rate-limiting enzyme in Heme catabolism [46]. NQO1 catalyzes the two-electron reduction of electrophilic quinone compounds, thus limiting the formation of semi-quinone radicals through one electron reduction and the subsequent generation of ROS [47]. Several polyphenols present in citrus and grapefruits have been reported to induce HO-1 expression in several cells [32,48,49]. Our experiments showed that the MIX 1:1 induced a significant increase in their expression, particularly in cardiomyocytes. Therefore, the coordinated action of radical scavenger and metabolizing enzymes ensure effective detoxification of ROS and contribute to the protective effects against the doxorubicin cardiotoxicity. 

As stated before, doxorubicin mediated apoptosis plays an important role in its cardiotoxicity, by activating both intrinsic and extrinsic pathways. Interestingly, seems that apoptosis process is linked to intracellular H_2_O_2_ formation and p53 protein activation in a different manner between healthy and cancer cells [50]. In this regard, it has been reported that *Citrus aurantifolia* ethanolic extract increase apoptosis induction on MCF-7 cells synergistically with doxorubicin, through regulation of protein p53 and Bcl-2 expression [51]. In addition, grape extract, besides its ability to suppress apoptotic process in cardiomyocytes [38], exert a pro-apoptotic function on MCF-7 by a mechanism that involves a transient increase of gap junction intracellular communication [52]. Therefore, the combination of orange and grapes phytochemicals can further strengthen both their cardioprotective potential and the synergistic activity with doxorubicin. In accordance with these considerations, we found that MIX 1:1 reduced apoptosis more in cardiomyocytes than in MCF-7 cells.

## 5. Conclusions

The data collected in the present paper highlight that smoothies represent an interesting option as onconutraceuticals, in particular when properly mixed in order to exert synergistic effect. In fact, consumption of smoothies could be useful not only in chemoprevention, but, in particular, as effective adjuvants for chemotherapy protocols. Despite further investigation being clearly needed, particularly in vivo, the in vitro results collected so far appear really promising.

## Supplementary Materials

The following are available online at https://www.mdpi.com/2076-3921/9/5/378/s1, Figure S1: MS (top) and MS/MS (bottom) spectra of (**a**) apigenin 6,8-C-β-d-glucopyranoside, (**b**) narirutin, (**c**) hesperidin, (**d**) nomilinic acid glycoside and (**e**) deacetyl-nomilinic acid glycoside; Figure S2: Effect of orange and red grapes extracts, and the 3 relative mixtures (25–1 μg/mL) alone on viability of H9c2 (**a**,**b**) and MCF-7 (**c**,**d**) cells. Data are expressed as mean ± S.E.M. of % of cell viability; Table S1: Qualitative profile of polyphenols isolated in *Citrus sinensis* smoothie; Table S2: RP-UHPLC-UV/Vis-ESI-IT-TOF identification of anthocyanins isolated from *Vitis vinifera L.* cv. (Aglianico N.) smoothie.
