# Supplementary material for: Citrus sinensis and Vitis vinifera Protect Cardiomyocytes from Doxorubicin-Induced Oxidative Stress: Evaluation of Onconutraceutical Potential of Vegetable Smoothies
Source: Antioxidants (Basel). 2020 May 2;9(5):378. doi: 10.3390/antiox9050378 (PMC7278676; doi:10.3390/antiox9050378)
Supplement: Supplementary file 1 [file antioxidants-09-00378-s001.pdf]

antioxidants

Supplementary Materials

Citrus sinensis and *Vitis vinifera* protect cardiomyocytes from doxorubicin-induced oxidative stress: evaluation of onconutraceutical potential of vegetable smoothies

Giacomo Pepe [1], Emanuela Salviati [1,2], Shara Francesca Rapa [1], Carmine Ostacolo [3], Stella Cascioferro [4], Michele Manfra [5], Giuseppina Autore [1], Stefania Marzocco [1,*] and Pietro Campiglia [1,6*]

[1] Department of Pharmacy, University of Salerno, Fisciano, Italy; gipepe@unisa.it (G.P.); esalviati@unisa.it (E.S.); srapa@unisa.it (S.F.R.); autore@unisa.it (G.A.); smarzocc@unisa.it (S.M.); pcampiglia@unisa.it (P.C.)
[2] PhD Program in Drug Discovery and Development, University of Salerno, Fisciano, Italy
[3] Department of Pharmacy, University of Naples Federico II, NA, Italy; ostacolo@unina.it
[4] Dipartimento di Scienze e Tecnologie Biologiche, Chimiche e Farmaceutiche (STEBICEF), University of Palermo, PA, Italy; stellamaria.cascioferro@unipa.it
[5] Department of Science, University of Basilicata, Viale dell'Ateneo Lucano 10, I-85100 Potenza, Italy; michele.manfra@unibas.it
[6] European Biomedical Research Institute of Salerno, SA, Italy
* Correspondence: smarzocco@unisa.it (S.M.); pcampiglia@unisa.it (P.C.)
Tel.: +39-089-96-9250 (S.M.); +39-089-96-9242 (P.C.)

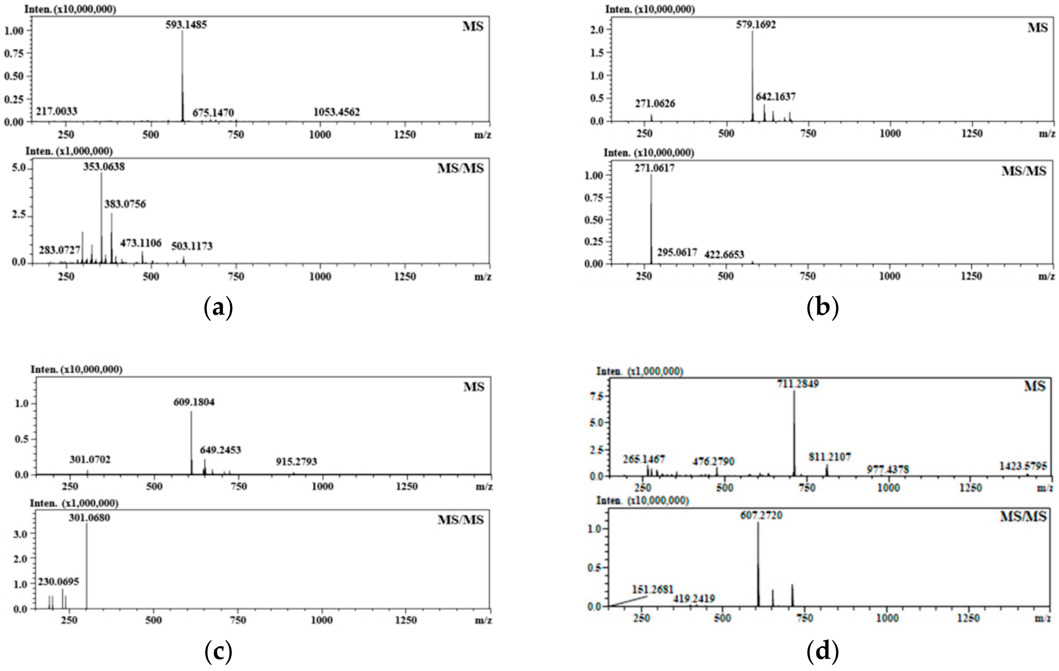

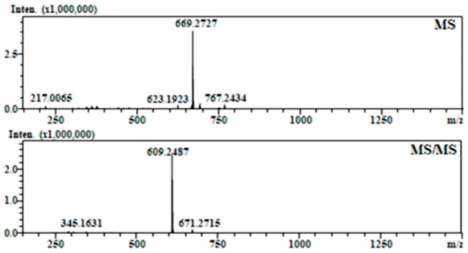

(**e**)

Figure S1. MS (top) and MS/MS (bottom) spectra of (**a**) apigenin 6,8-C-β-D-glucopyranoside, (**b**) narirutin, (**c**) hesperidin, (**d**) nomilinic acid glycoside and (**e**) deacetyl-nomilinic acid glycoside.

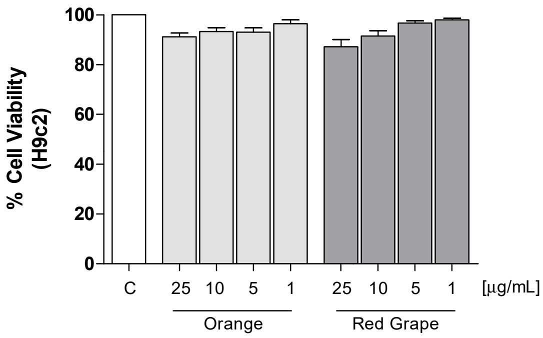

(**a**)

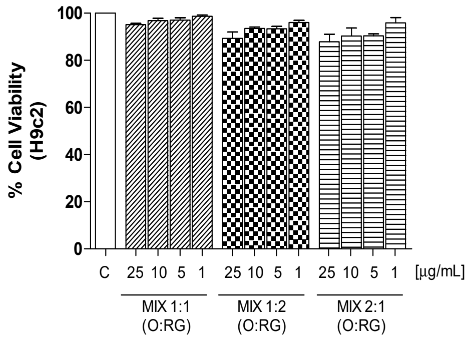

(**b**)

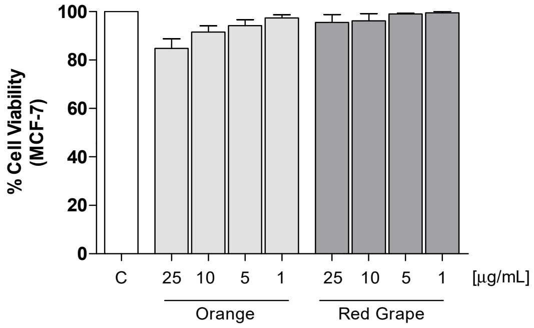

(**c**)

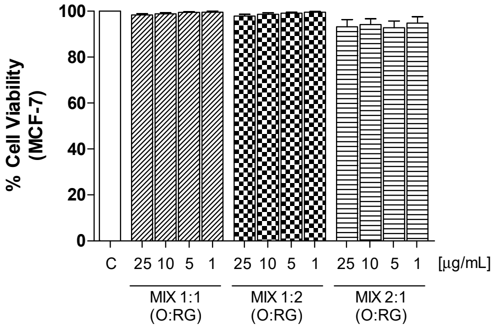

(**d**)

Figure S2. Effect of orange and red grapes extracts, and the 3 relative mixtures (25 – 1 µg/mL) alone on viability of H9c2 (**a, b**) and MCF-7 (**c, d**) cells. Data are expressed as mean ± S.E.M. of % of cell viability.

Table S1. Qualitative profile of polyphenols isolated in *Citrus sinensis* smoothie.

Peak	Rt (min)	Molecular Formula	Compound	[M-H]-	MS/MS	Error (ppm)
1	1.20	$C_7H_{12}O_6$	quinic acid	191.0241	173.0132	-5.76
2	1.21	$C_{16}H_{19}O_9$	caffeoylquinic acid	353.0736	173.0147	-2.35
3	4.52	$C_{15}H_{18}O_8$	5-p-coumaroyl-hexoside acid	325.0945	163.0311, 191.0570	-4.92
4	5.30	$C_{16}H_{20}O_9$	ferulic acid 4-*O*-glucoside	355.1026	175.0549, 193.0527,	-2.53
5	5.55	$C_{16}H_{18}O_{11}$	ferulic acid hexoside	385.0757	191.0102	-4.93
6	5.65	$C_{33}H_{40}O_{21}$	quercetin 3-*O*-glucosyl-rhamnosyl-hexoside	771.2018	301.0359, 609.1374, 271.0196	-4.28
7	6.75	$C_{27}H_{30}O_{15}$	vicenin II (qpigenin 6,8-C-*β*-D-glucopyranoside)	593.1466	503.1173, 473.1107, 353.0692,383.0805,	2.19
8	7.15	$C_{21}H_{22}O_{10}$	naringenin hexoside	433.1130	271.0631,	-0.85
9	7.87	$C_{28}H_{32}O_{16}$	lucenin 2-4′-methyl-ether	623.1581	383.0751, 312.0561	3.70
10	9.04	$C_{27}H_{32}O_{15}$	eriocitrin	595.1631	287.0542	-1.96
11	9.29	$C_{33}H_{40}O_{21}$	quercetin 3-*O*-glucosyl-rhamnosyl-hexoside	771.2360	301.0704, 463.1202	-4.28
12	9.32	$C_{21}H_{20}O_{12}$	quercetin-3-*O*-hexoside	463.1342	301.0706	3.81
13	10.05	$C_{26}H_{28}O_{14}$	apigenin 7-*O*-apiosyl-glucoside	563.1366	293.0344, 413.0874	-4.90
14	10.75	$C_{27}H_{30}O_{16}$	rutin	609.1472	301.0335, 271.0237	-3.89
15	11.63	$C_{27}H_{32}O_{14}$	narirutin	579.1703	271.0590	-4.19
16	12.25	$C_{33}H_{40}O_{19}$	naringenin 3-*O*-glucosyl-rhamnosyl-hexoside	739.2122	433.1115, 577.1521, 271.0605	-2.22
17	12.76	$C_{27}H_{32}O_{14}$	naringenin 7-*O*-neohesperidoside	579.1688	271.0669, 357.0802	-1.51
18	13.85	$C_{28}H_{34}O_{15}$	hesperidin	609.1798	301.0679	-4.43
19	14.07	$C_{32}H_{42}O_{14}$	limonin glucoside (limonin 17*β*-*D*-glucopyranoside)	649.2479	443.2036, 605.2564,651.2506	-1.45
20	14.44	$C_{28}H_{32}O_{16}$	isorhamnetin 3-*O*-rutinoside	623.1591	315.0512, 311.0222, 271.0239	-2.35
21	15.19	$C_{28}H_{34}O_{15}$	hesperidin isomer	609.1782	301.0702	-6.37
22	15.50	$C_{28}H_{34}O_{15}$	hesperidin isomer	609.1798	301.0785	-6.37
23	17.70	$C_{39}H_{42}O_{10}$	deacetyl-nomilinic acid glycoside	669.2720	609.2463	2.24
24	20.59	$C_{28}H_{34}O_{14}$	didymin	593.1848	285.0772	2.72
25	21.90	$C_{34}H_{46}O_{15}$	nomilin glucoside	693.2736	565.2589, 395.1648,445.2121	-4.04
26	21.92	$C_{34}H_{48}O_{14}$	nomilinic acid-glycoside	711.2834	607.2710	-3.80

Table S2. RP-UHPLC-UV/Vis-ESI-IT-TOF identification of anthocyanins isolated from *Vitis vinifera* L. *cv. Aglianico N.* smoothie.

Peak	Rt (min)	Molecular Formula	Compound	$[M+H]^+$	Error (ppm)
1	4.34	$C_{21}H_{21}O_{12}^+$	delphinidin 3 *O*-glucoside	465.1037	1.94
2	4.78	$C_{21}H_{21}O_{11}^+$	cyanidin 3 *O*-glucoside	449.1095	5.57
3	5.13	$C_{22}H_{23}O_{12}^+$	petunidin 3 *O*-glucoside	479.1202	3.76
4	5.58	$C_{22}H_{22}O_{11}^+$	peonidin 3 *O*-glucoside	463.1258	4.97
5	5.86	$C_{23}H_{25}O_{12}^+$	malvidin 3 *O*-glucoside	493.1349	2.50
6	7.32	$C_{24}H_{24}O_{13}^+$	petunidin 3 *O*-(6″acetyl) glucoside	521.1238	3.75
7	7.29	$C_{24}H_{24}O_{13}^+$	peonidin 3 *O*-(6″acetyl) glucoside	505.1365	4.07
8	7.77	$C_{24}H_{24}O_{13}^+$	malvidin 3 *O*-(6″acetyl) glucoside	535.1493	1.96
9	8.29	$C_{32}H_{30}O_{15}^+$	malvidin 3 *O*-(6″-p-caffeoyl) glucoside	655.1943	3.78
10	8.60	$C_{31}H_{28}O_{14}^+$	petunidin 3-(6-coumaroyl) *O*-glucoside	625.1587	2.44
11	9.10	$C_{31}H_{28}O_{13}^+$	peonidin 3-(6-coumaroyl) O-glucoside	609.1658	3.89
12	9.19	$C_{32}H_{30}O_{14}^+$	malvidin 3 *O*-(6″-p-coumaroil) glucoside	639.1747	4.67
